# Investigating the effect of chitosan, nanopackaging, and modified atmosphere packaging on physical, chemical, and mechanical properties of button mushroom during storage

**DOI:** 10.1002/fsn3.1294

**Published:** 2019-12-05

**Authors:** Rashid Gholami, Ebrahim Ahmadi, Shervin Ahmadi

**Affiliations:** ^1^ Department of Biosystem Engineering Faculty of Agriculture Bu‐Ali Sina University Hamedan Iran; ^2^ Iran Polymer and Petrochemical Institute Tehran Iran

**Keywords:** button mushroom, engineering properties, packaging, shelf life

## Abstract

This study investigated the effect of chitosan coating, nanopackaging, and modified atmosphere packaging (MAP) to increase the shelf life and improve the quality of the white button mushroom. Uncoated and coated button mushrooms were stacked in three types of packages (normal, nano, and nano + MAP). The atmosphere inside the nano + MAP packages contained 10% oxygen and 10% carbon dioxide. The physical, chemical, mechanical properties and the evolution of oxygen and carbon dioxide inside the packages were investigated. The results showed that the triple interaction had significant effect (at 1% and 5% levels) on physical, mechanical properties and gas composition. The use of nanofilm (due to the low permeability to oxygen and carbon dioxide), as well as the modification of atmosphere had a positive effect on the control of mushroom respiration rate and the improvement in its physical, chemical, and mechanical properties.

## Graphical abstract

The results of this study showed that the using of nano film and nano + MAP condition had positive effects on preserving physical, chemical, and mechanical properties of white mushroom during storage and also can extend the shelf life of mushroom. Therefore, using of nano film along with MAP is recommended for using in agricultural products packaging and food packaging industry especially for sensitive products like mushrooms.

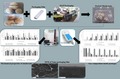

## 1 INTRODUCTION

White button mushroom (*Agaricus bisporus*) is known as the most popular and most commonly used mushroom in the world, followed by shiitake and straw mushrooms that are, respectively, ranked as the second and third most popular mushrooms (Gholami, Ahmadi, & Farris, 2017; Graham, Murrell, & Wai‐Kit, 2004). In 2017, the estimated rate of mushroom produced in Iran was 76,893 tons; as a result, Iran is recognized as one of the most important sources of mushroom production (Anonymous, 2017). The shelf life of button mushroom is about 3–4 days in ambient temperature without any packaging, which makes button mushroom highly putrescible as compared with many edible and fresh agricultural products; this leads to high wastes after harvesting because it lacks cuticle to protect it from physical damage and microbial attack (Donglu et al., 2016). Among the factors that contribute to the short life of button mushroom, the enzymatic activity, browning, moisture loss, microbial attacks, and very high respiration rate are the most important ones. Therefore, button mushroom requires special care and special packaging at postharvest stages. Many methods have been proposed to increase the shelf life of edible mushroom. The use of nanocomposite films, the modification of the atmosphere inside product packages, the use of edible coatings, and the control of product storage temperature seem to be the most important and efficient methods (Kim, Ko, Lee, Park, & Hanna, 2006; Roy, Anantheswaran, & Beelman, 1995; Tripathi, Mehrotra, & Dutta, 2009).

Chitosan is a natural polymer obtained from chitin, which is abundantly found in crustacean shells (crabs and shrimp; Liu, Liu, Zhang, Kan, & Jin, 2019). Chitosan is nontoxic, biodegradable, and environmentally friendly. Because of its antimicrobial properties, it restricts the growth of many bacteria. For these reasons, chitosan has been extensively used as an edible coating in fruits and vegetables (Tripathi et al., 2009). Improving the quality and shelf life of sliced pears by using the coating (Xiao, Zhu, Luo, Song, & Deng, 2010), and the use of chitosan in preserving the quality and properties of strawberries (Munoz, Almenar, Valle, Velez, & Gavara, 2008). Also some studies showed that chitosan can preserve the taste of pear fruit, which after cold storage was similar to the taste of the fresh fruit (Zhou et al., 2008).

Nanotechnology has been proposed as one of the most promising strategy to improve the overall properties of food packaging materials, thus extending the shelf life of packaged food (Uysal Unalan, Cerri, Marcuzzo, Cozzolino, & Farris, 2014). Both nanocomposite and nanostructured materials (and a combination thereof) have been proposed to this scope (Fuentes‐Alventosa et al., 2013). Different types of nanoparticles have been used to improve packaging films. The increasing use of nanoparticles in the production of food and agricultural nanocomposite packages is important because it not only reduces environmental problems but also improves the performance of these materials during the storage time (Taghizadeh, Gowen, Ward, & O'Donnell, 2010). Nanoclays are relatively new materials that are incorporated into compounds of packaging films. Polymer–clay nanocomposite is an alternative to traditional polymers; they are produced in a nanosize that improves their mechanical and physical properties, permeability, and thermal stability (Abdollahi, Rezaei, & Farz, 2012; Tornuk, Hancer, Sagdic, & Yetim, 2015). The use of nanoparticles as fillers or additives has become very popular in recent years. For example, Tornuk et al. (2015) investigated the use of a linear low‐density polyethylene (LLDPE) equipped with nanoclay particle for improving and prolonging the shelf life of some meat products. The results showed that fresh beef color was maintained up to 4 days by active nanocomposite film. They reported that in conclusion, it might be suggested that active clay nanocomposite packaging film could be used to extend shelf life of the fresh and/or processed meats. Li et al. (2009) investigated the maintenance of the quality of Chinese jujube during the storage time using nanocomposite packaging. The results showed that this nanocomposite had a quite beneficial effect on physicochemical and sensory properties of this product compared with normal packing material during the storage time. Effect of nanocomposite packaging material (Nano‐PM) on physicochemical characteristics and antioxidant capacity of mushrooms (*Flammulina velutipes*) during storage time at 4°C was investigated by Donglu et al. (2016). The results showed that Nano‐PM improved retention of nutrients and inhibited the weight loss, respiration, and distinct stipe elongation of mushrooms compared with the normal packing material.

Modified atmosphere packaging (MAP) of food and agricultural products is also used as a method to increase the product shelf life and divided into two methods, passive and active. Passive MAP, where effects are derived from product respiration rate and gas permeability of the packaging film, induces a passively established steady state after a long transient period. With active MAP, initial gas flushing or the introduction of a gas scavenging system within the package is used to accelerate gas composition modification to avoid product exposure to high concentrations of unsuitable gases (Charles, Guillaume, & Gontard, 2008). According to this approach, MAP aims to reduce the oxygen and increase the carbon dioxide concentrations inside the package to increase the shelf life of the product, through reducing the respiration rate and the metabolic activity of the product, as well as delaying the enzymatic activity (Jiang et al., 2011). Also, MAP does not include direct chemical reactions or the use of preservatives, which are strictly controlled by the EU regulative (Djekic et al., 2017). The composition of the atmosphere surrounding the product (i.e., inside the package) is changed depending on the properties of the product (Ares, Lareo, & Lema, 2007). The ranges of 3%–21% for oxygen and 5%–15% for carbon dioxide were recommended for mushrooms by Sandhya (2010).

### 1.1 Literature review of postharvest quality and shelf life of mushrooms

Some of researches that have been done about mushrooms presented in this section. Use of coatings for improving and preserving physical, mechanical, and chemical properties of Shiitake mushroom was investigated by Jiang, Feng, and Li (2012). On that research application of chitosan, glucose and chitosan–glucose complex were used as shiitake mushroom coating. Weight loss, respiration rate, firmness, ascorbic acid, total soluble solids, and microbial and sensory quality of samples were measured during 16 days of storage. The results indicate that treatment with chitosan–glucose complex coating maintained tissue firmness, inhibited increase of respiration rate, reduced microorganism counts, for example, pseudomonads, yeasts, and molds, compared to uncoated control mushroom.

Oliviera, Sousa‐Gallagher, Mahajan, and Teixiera (2012) investigated the modified atmosphere packaging of sliced mushrooms, and the results of their study showed that the optimum condition for storing the mushroom was achieved by a gas mixture of 3.6% oxygen and 11.5% carbon dioxide. Moreover, according to Villaescusa and Gil (2003) the best gas mixture for storing some types of edible mushroom consisted of 15% oxygen and 5% carbon dioxide.

The effects of polyvinyl chloride (PVC), silicon windows (SW), and polyethylene (PE) packaging materials on the sensory of texture, senescence, browning, and odor changes of pine mushroom have been evaluated, and the preliminary mechanisms have been studied by Wei et al., (2017). Their results showed that texture changes were most efficiently delayed by PE as a result of the lowest respiration rates and weight loss. Senescence was most efficiently delayed by PE and PVC which may be related with the low O_2_ and CO_2_ transmission properties of PE and PVC packaging.

Effect of gallic acid grafted chitosan film packaging on the postharvest quality of white button mushroom (*Agaricus bisporus*) investigated by Liu et al. (2019). The results of their research showed that, as compared to mushrooms packaged with chitosan film and commercially used polyethylene film, mushrooms packaged with gallic acid grafted chitosan film showed significantly lower respiration rate, browning degree, malondialdehyde content, electrolyte leakage rate, superoxide anion production rate, and hydrogen peroxide content. Their results suggested gallic acid grafted chitosan film packaging could increase the antioxidant status of *A. bisporus*, which in turn maintained the postharvest quality of mushrooms.

A literature survey on the investigations related to the shelf life extension of white button mushroom showed that the effects of chitosan coating, some of nanofilms, and modified atmosphere packaging on the product have already been investigated, however, individually. That is, so far no research has been focused on the concurrent use of these methods and their effects on the properties of this product during the storage time. The aim of this study was therefore to determine the effect of three different approaches (edible coating, nanocomposite packaging film, and MAP) on the overall properties (physical, chemical, and mechanical) of white button mushroom during storage time. This research involves two types of original features: first, it used a combination of existing methods to improve the quality of white button mushroom during the storage time; second, it involved the use of low‐density polyethylene (LDPE) packaging film loaded with nanoclay particles for packaging this product, which has never been reported before.

## 2 MATERIALS AND METHODS

### 2.1 Plant material

White button mushrooms were cautiously handpicked from Sadra Mushroom Cultivation Industry Company in Hamedan city. Mushrooms were selected at their maturity stage, with a completely closed cap, and almost identical in shape. The samples were transferred to the laboratory and stored at a temperature of 4 ± 1°C and a humidity of 75 ± 2% for 24 hr in order to stop the growth before the start of the experiments.

### 2.2 Preparation of chitosan solution and packaging

Chitosan powder (degree of deacetylation 75%–85%; molar mass distribution: 190,000–310,000; viscosity range: 200–800 cP, 1 wt % in 1% acetic acid at 25°C by Brookfield method) was obtained from Sigma Aldrich Company. A solution of 1% chitosan was prepared by dissolving 5 g of chitosan powder in distilled water plus acetic acid at a pH = 4 (Maghsudlo, Maghsudlo, Khamiri, & Ghorbani, 2012; Yingyuad et al., 2006) and homogenized for 2 min; finally, the pH of chitosan solution was 5.5. The dipping method was used to coat the samples; first, mushrooms, one by one, were placed into the chitosan solution for 1 min and then placed under ambient air for 2 hr in order to lose excess moisture and water (Ghasemi Tvallaei, Ramin, & Amini, 2015; Kim et al., 2006). After coating deposition, coated and uncoated mushrooms (100 g) were separately packed using three different packaging materials: (a) polyvinyl chloride (PVC), 11 µm thick, as a conventional packaging material; (b) LDPE nanocomposite film with atmosphere gas composition; and (c) LDPE nanocomposite film in combination with MAP. Packaged mushrooms were stored at 4 and 25°C. All the experiments were carried out over a period of 10 days every other day.

### 2.3 Preparation of nanoclay film

Ethylene‐vinyl alcohol (EVOH; containing 32% ethylene) with a melt flow index (MFI) of 3.8 g/10 min and a density of 1.19 g/cm^3^ was purchased from the Soarnol Company. Low‐density polyethylene (LDPE) with a grade of 0020 was used with a MFI of 2 g/10 min and a density of 0.920 g/cm^3^. Polyethylene‐grafted maleic anhydride (LDPE‐g‐MA) with a melt flow index of 1.2 g/10 min and a density of 0.923 g/cm^3^ was purchased from Karnegin Company.

The melted mixture was prepared in a twin‐screw extruder (a Barbender co‐rotating twin‐screw extruder model 2002, manufactured in Germany with L/D = 40) at a speed of 100 rpm and at a temperature range of 180–220°C. Initially, LDPE/Cloisite^®^ 20A containing 8% wt LDPE mixed with LDPE/LDPE‐g‐MA containing 20% wt LDPE‐g‐MA. Finally, the film was produced through impact method and single‐screw extrusion (Brabender) with L/D = 26 at a temperature range of 180–220°C and speed of 100 rpm. The thickness of the produced film was 100 ± 5 μm. Finally, the film containing 10% LDPE‐g‐MA, 2% Cloisite^®^ 20A (nanoclay), and 10% EVOH was prepared (Nooshirvani, Ghanbarzadeh, & Entezami, 2011; Rahnama, Oromiehie, Ahmadi, & Ghasemi, 2016).

### 2.4 Properties of nanofilm

The oxygen and carbon dioxide transmission rate (OTR and CO_2_TR, respectively) were measured using GDP‐c gas permeability tester (Coesfeld Meteriatest) based on ASTM D3985 and ASTM F1927 standard methods. A circle with a diameter of 150 mm and a thickness of 100 micrometers was provided for testing. Tests were carried out at 23°C and at two different RH conditions, that is, 0% and 65% RH.

Morphological properties of the film were investigated using Scanning Electron Microscopy (SEM; Tescan). An electron microscope with an electric current of 3 A and a voltage of 15 kV at a pressure of 5 Pa was used. In order to extract EVOH, the samples were coated with dimethyl sulfoxide for 4–6 hr at a temperature of 50°C, then dried at a temperature of 50°C for 24 hr and finally were coated with gold, and their cross sections were used as the samples.

The dispersion level and the orientation of nanoclay particles depend considerably on the molecular structure of intercalating polymers and their interactions with nanoclay (Ben Dhieb, Jalalidil, Tabatabaei, Mighri, & Ajji, 2019). In nanopackaging film permeability against O_2_, CO_2_ and WV (Water Vapor) were controlled because of the nanoclay plates structure (Zadeh, Seif, & Kadivar, 2015). The nanoparticles are located within the film structure and control the passage of gases; therefore, the permeability of the film will control, too. For nanofilm, OTR at 0% and 65% RH were 9.8 and 10.5 (cm^3^ STP/m^2^ 24 hr), respectively. CO_2_ transfer rate at 0% and 65% RH were 22.9 and 23.6 (cm^3^ STP/m^2^ 24 hr), respectively, and WV transfer rate was 2.5 (g/m^2^ 24 hr) at 90% RH. While for PVC, OTR was more than 7,500 (cm^3^ STP/m^2^ 24 hr), CO_2_ transfer was more than 18,000 (cm^3^ STP/m^2^ 24 hr), and VW transfer was 148.47 (g/m^2^ 24 hr). Similar results about the effects of nanoparticles on permeability of packaging were reported, too. Using of nanoparticles like nanoclay and nanosilver have effects on impermeability of packaging film as Sattari, Minaii, and Azizi, and Afshari (2010) have mentioned. The results showed that the changes in moisture did not have a significant effect on the changes in oxygen and carbon dioxide permeability of the film. The permeability of the film to oxygen and carbon dioxide gases, as well as to the water vapor, is very low. The low permeability of the film to the oxygen and carbon dioxide gases prevents the entry of these gases into the package; it has a remarkable impact on controlling the respiration rate of products packed with this type of film. The low permeability of the film to water vapor also has a positive effect on maintaining the moisture content of the product packed with this type of film.

**Figure 1:**
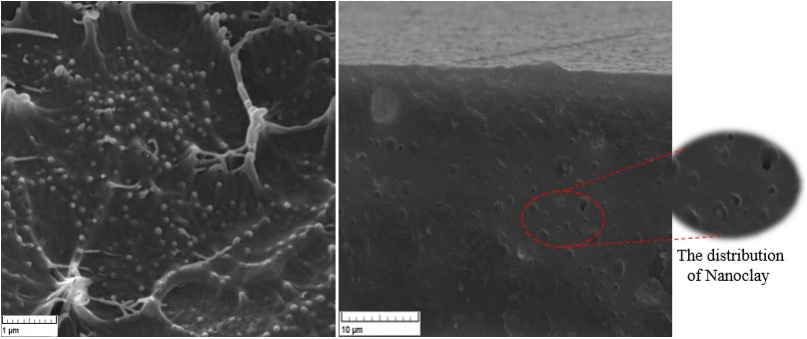
SEM images of (a) surface and (b) cross section of polyethylene film equipped with Nanoclay

The SEM images of the LDPE film loaded with nanoclay particles and its cross section are shown in Figure 1. The images show the uniform distribution of nanoclay which is the main reason for the improved permeability and mechanical properties of the nanocomposite films, as compared with the conventional films.

### 2.5 Composition of gas inside the packages and its analysis

In this study, the packages made with nanofilm were filled with a specific percentage of gases (provided by a capsule containing 10% O_2_ + 10% CO_2_). The sealed packages 15 cm × 20 cm × 7 cm were prepared by using semi‐automatic seal vacuum system (STS007, West Asia Steel Co). Oxygen and carbon dioxide were measured every 2 days (for 10 days) using a gas meter (Oxybaby 6i, WTT‐GASTECHNIK).

### 2.6 Mechanical properties

In order to evaluate the mechanical properties of the sample, penetration test was carried out using food test apparatus (Bbt1‐Fro.5th.D14, Zwick‐Roell) equipped with a load cell (X Force Hp nominal Force: 500 N Capacity). To carry out the test, a probe of 5 mm diameter with a speed of 10 mm/min was used (Gholami et al., 2017). In this test, the probe penetrated into the samples to a given depth and recorded the elastic modulus and maximum force (known as firmness of texture) to penetrate the product. Three replicates for each treatment were performed.

### 2.7 Physical properties

The percentage of weight loss of the samples was determined by weighing the samples in each packages at the beginning and during the storage time at specified time intervals using Equation 1. The packages were weighed using a digital scale with an accuracy rate of 0.01 g.

(1) $$\text{Weight}\;\text{loss}=\frac{W_{0}-W_{1}}{W_{0}}\times 100$$

where *W*_0_ is the initial weight of the samples, and *W*_1_ is the weight of each sample at a given day.

The color of the surface of the mushroom cap (as the most important part of the mushroom) was tested in the laboratory using a digital portable color meter model hp‐200 (Shenzhen Handsome Technology Co., Ltd.). To perform this test, three samples out of each treatment were randomly selected and then numbered. A specific area on the product was marked by a marker and the color changes at that area was recorded during the storage time. All the samples were tested by the color tester and the CIE L*a*b* coordinates were recorded. The overall color variation (Δ*E*) was calculated using the following equation (Islam, Zhang, Adhikari, Xinfeng, & Xu, 2014; Oliviera et al., 2012):

(2) $$\Delta E=\sqrt{\Delta L^{{*}^{2}}+\Delta a^{{*}^{2}}+\Delta b^{{*}^{2}}}$$

In addition, the following equation was used to calculate browning index, which is an important parameter in products with enzymatic activity (Oliviera et al., 2012):

(3) $$\text{BI}=\frac{100\left(x-0.31\right)}{0.172}$$

(4) $$x=\frac{a+1.75L}{5.645L+a-3.012b}$$

### 2.8 Percent open caps

One of the criteria for checking the quality of white button mushrooms is the development and opening of caps, forming an umbrella‐shaped cap. Cap opening is one of the determinants of senescence and the loss of mushroom quality, which is associated with changes in mushroom color and directly affects the marketability of the product. The following equation and 5–6 number of samples were used to calculate the percentage of cap opening for each situation during the storage time:

(5) $$\text{Percent}\;\text{open}\;\text{caps}\left(\%\right)=\frac{N_{O}}{N_{t}}\times 100$$

where *N*_t_ is the total number of samples per package, and *N*_O_ is the number of opened caps in the same package.

### 2.9 Chemical properties

In order to measure pH and total soluble solid (TSS), mushrooms were first ground in a mortar and homogenized, and their extract was obtained with a handpress. The obtained extract was filtered by a 40 μm filter paper. The pH of the extract was measured using a pH meter with a resolution of 0.01 (PHS3‐WB, Bante). TSS was measured at 25°C using the Atago refractometer (PAL‐2) with a resolution of 0.01. Each test was done with three replications.

### 2.10 Statistical analysis

The effect of chitosan coating, packaging method, and storage time was investigated as parameters affecting the physical, chemical, and mechanical properties of button mushroom. Each experiment was done in three replications by using three different samples. Statistical analysis was done by factorial test in completely randomized design. The data obtained from the experiments were statistically analyzed using the ANOVA test. In addition, the comparison of the means was done by using Duncan method, and the probability value of 1 percent was considered. The analyses were performed in SPSS 19 software, and the charts were drawn by Excel 2013 software.

## 3 RESULTS AND DISCUSSION

### 3.1 Study of packaging conditions and the characteristics of mushroom

All packages were divided into two groups and stored at 4 and 25°C. Samples stored at a temperature of 25°C had become completely corrupted and unusable after 4 days. Therefore, the results of analyses presented in this paper are related to samples stored at 4°C that were stored for 10 days at this temperature.

### 3.2 Evaluation of gas composition during the storage time

**Figure 2:**
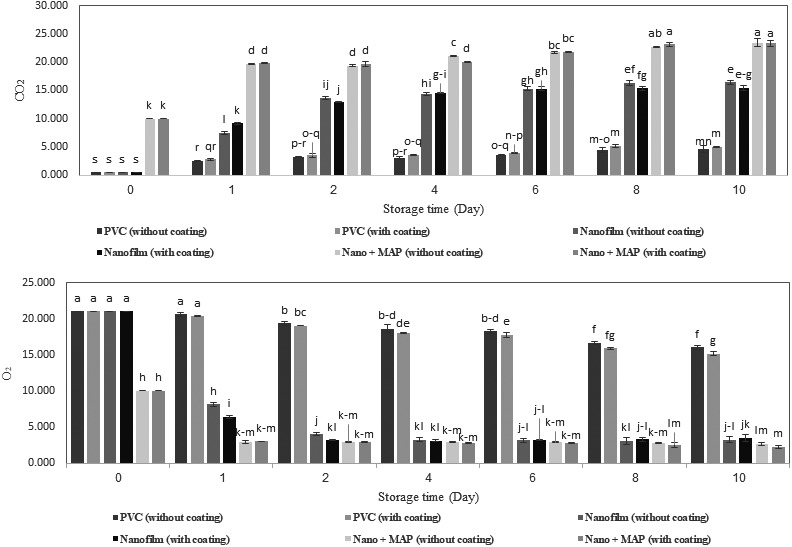
Changes in the amount of CO_2_ and O_2_ inside the package during the storage time

The results of analysis of variance (Table 1) showed that the storage time, type of packaging, the double interaction effects time × packaging, and coating × packaging, and the triple interaction effects of time × coating × packaging had significant effects on the changes in oxygen and carbon dioxide content inside the packages at a significance level of 1%. However, chitosan coating did not have a significant effect on changes in carbon dioxide. We compared the mean values of the main effects, and the results showed that the amount of gases in each of the three types of packaging was significantly different from each other. The significant difference between conventional packaging and nanopackaging in terms of the amount of oxygen and carbon dioxide was attributed to the resistance of nanofilm against the entering and exiting gases as also mentioned by Zadeh et al. (2015). Moreover, the significant difference between nano + MAP packaging and nano and conventional packaging was attributed to the initial composition of the gases inside the nano + MAP packages. The changes in oxygen inside the packages with conventional film (23.76%) were less than the changes in oxygen in nanofilm packages (84.58%) and in nano + MAP packages (73.33%); it can be attributed to the high permeability of conventional film (PVC) to oxygen that, concurrent with the respiration of the product inside the package, causes gas exchange between the inside and outside gases. However, because of the low permeability of the nanofilm packages, the transfer of gas from outside into the package is very low, and because of the respiration of the product, there is a sharp drop in oxygen content and an increase in carbon dioxide levels inside the package (Figure 2). The difference between gas composition inside PVC and nanopackages is related to film permeability while in nano + MAP, permeability and the first gas composition, which was injected inside packages, both had effect. The assessment of the mean effects of storage time on the changes in oxygen and carbon dioxide showed that the changes in carbon dioxide between the 8th and 10th day, as well as the changes in oxygen content between the 4th and 6th day were not significant. However, in other days we observed significant differences between the effects of the storage time on mean values at a significance level of 1%.

**Table 1:** Analysis of variance of the effect of storage time, chitosan coating, and type of packaging on the gas composition and mechanical properties of button mushroom

Source changes	*df*	*E*_mod_	*F*_max_	CO_2_	O_2_
Storage time	5	0.00**	5.40**	51.30**	21.63**
Coating	1	0.06**	0.19^ns^	0.21^ns^	3.72**
Packaging	2	0.02**	35.57**	2,585.61**	2,570.64**
Storage time × coating	5	0.00^ns^	3.10^ns^	0.50**	0.12^ns^
Storage time × packaging	10	0.00^ns^	2.78^ns^	9.75**	6.67**
Coating × packaging	2	0.00**	12.30**	0.79**	0.44**
Storage time × coating × packaging	10	0.00*	3.43*	0.75**	0.51**
Error	72	0.00	1.80	0.08	0.06

Abbreviations:* E*_mod_, Module of elasticity; *F*_max_, Maximum force; ns, No significant difference.

** A significant difference in the level of 1%.

* A significant difference in the level of 5%.

Figure 2 presents the changes in oxygen and carbon dioxide content and the interaction effect of time × coatin × packaging on the mean values of these gases. The results showed that in all the treatments, due to the respiration of the product inside the package, with the passage of time the amount of oxygen decreased and the amount of carbon dioxide increased. The study of the interaction effect of time × coating × packaging on oxygen and carbon dioxide content showed that the lowest amount of oxygen and the highest amount of carbon dioxide at the end of the storage time were occurred in nano + MAP packages (both coated and uncoated samples) were 2.26% and 23.46%, respectively.

Mushrooms have high respiration rate, creating a modified atmosphere, and equilibrium was achieved quickly (Oliviera et al., 2012). The amount of oxygen in the nano and nano + MAP packages had become almost stable after the second day until the end of the storage time, and the amount of oxygen remained in a range of 3.23%–4.05% in nanopackages, which this range is between recommended value of oxygen for mushrooms (Sandhya, 2010), and in a range of 2.26%–2.96% in nano + MAP packages. In line with the results of our study, Oliviera et al. (2012) reported the changes in oxygen content of the packages containing sliced mushroom, so that Equilibrium gases composition in headspace was achieved after, approximately, 1 day (Oliviera et al., 2012). However, the amount of oxygen in conventional packages had a falling trend and the amount of carbon dioxide had rising trend until the end of the storage time, which was due to the high permeability of ordinary film. The results showed that the amount of oxygen and carbon dioxide was ≈16% and ≈5%, respectively, at the end of the storage time, which both of them are in recommended range that reported by Sandhya (2010), but should notice that, concurrent with the consumption of oxygen inside the package, the oxygen from the surrounding environment entered the package and prevented the reduction of respiration, which accelerated the spoilage of products packed with PVC.

### 3.3 Change in the firmness of texture during the storage time

**Figure 3:**
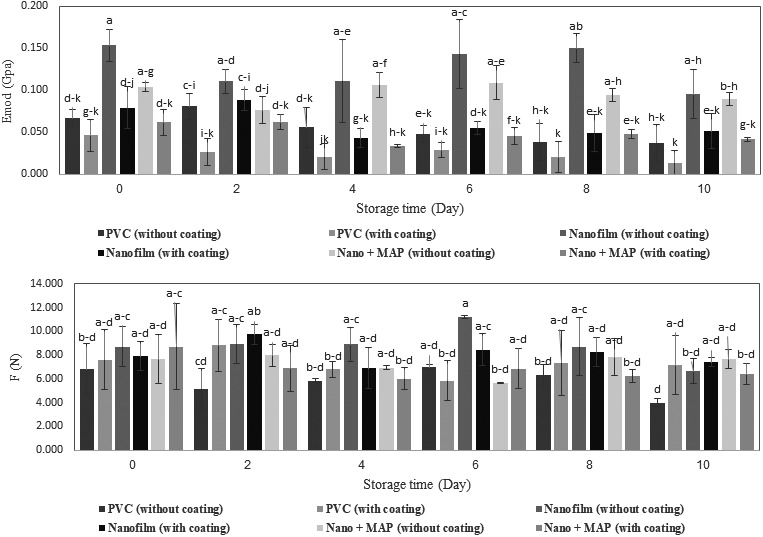
Changes in elastic modulus and *F*_max_ during the storage time

Mushroom texture is a major factor in determining the quality of mushrooms and has a direct impact on the metabolism and water content of this product. The increase in the shelf life of mushroom affects the firmness of the texture (Islam et al., 2014; Rux et al., 2015). Loss of firmness is caused by chitin synthesis in cell walls, leading to toughening; protein and polysaccharide degradation; and loss of cell turgency due to changes in cell membrane permeability, leading to softening of mushrooms (Oliviera et al., 2012). The results show the decrease in elastic modulus (*E*_mod_) and maximum force (*F*) for all the treatments during the storage time (Figure 3). The results of this study on button mushroom showed that the use of nano + MAP packaging was more effective than other methods in controlling texture firmness. The lowest level of change (decrease) in the elastic modulus (15.84%) and the penetration force (0.66%) was observed in products in nano + MAP packages. The same results about the effect of MAP on mechanical properties of *A. bisporus* were reported by Oz, Ulukanli, Bozok, and Baktemur (2015), too. The results of analysis of variance showed that except for the interaction effects of time × coating and time × packaging, other treatments (time, packaging, coating, and interaction effects) had a significant effect on the elastic modulus at a significance level of 1% and 5% (Table 1). In addition, time × packaging and time × coating × packaging had a significant effect on the changes in force. The trend of changes in force and elastic modulus during the storage time and the effect of the interaction effects of the time × coating × packaging on the mean values of these two factors are shown in Figure 3. The assessment of mean values showed that, except for some cases, the changes in the day of testing had a significant effect on the elastic modulus and penetration force. Consistent with our results, the significant effects of the storage time on the firmness of tomato texture were reported, too (Tabatabaei Kloor, Ebrahimian, & Hashemi, 2016). The results of the assessment of the mean values showed that there was no significant difference between nano + MAP packaging and conventional packaging in terms of penetration force; however, the elastic modulus had a significant difference between all the three packaging conditions. The softness and the loss of stiffness in fresh products have a direct relationship with the reduction of water content and product weight (Rux et al., 2015). Because of the spongy texture, the surface of button mushroom absorbs water in touch of each solution, so the use of chitosan coating had a detrimental effect on the texture of this product; therefore, a higher level of decrease in the elastic modulus in the coated samples than in uncoated samples was observed. The results of analysis of variance showed that the effect of chitosan coating on reducing the elastic modulus was significant, while there was no significant difference between the coated and uncoated samples in terms of penetration force.

### 3.4 Weight loss during the storage time

The weight loss during the 10 days of storage has increased in all the treatments. The results of analysis of variance of data showed that the type of packaging, storage time, and dual interaction effects of time × packaging significantly affected the changes in weight loss (Table 2). The acceptable and marketable weight loss level should be under 10% (Roy et al., 1995). At the end of the storage time, the highest level of weight loss was observed in packages with nanofilm and the lowest level of weight loss was observed in nano + MAP packages, which were 3.58% and 0.12%, respectively, so all types of packages were acceptable from point of view of weight loss. The results of the assessment of the comparison of the mean values showed that there was a significant difference between the three types of packaging, and nano + MAP packages have significantly controlled the weight loss of the samples. This result indicated that the use of specific percentages of gases (low amount of oxygen and high amount of carbon dioxide) in the package of button mushroom had a positive and significant effect on reducing the weight loss of this product during the storage time at a significance level of 1%. Using of CO_2_, inside packages can control the respiration and moisture of the samples, and consequently, weight loss was controlled too, as Mohammadpuor and Tajoddin (2017) have reported. Djekic et al. (2017) and Tabatabaei Kloor et al. (2016) reported similar results for mushrooms and tomatoes about the effect of high CO_2_ on controlling weight loss. The results of the assessment of the comparison of the means of the storage time on weight loss indicated an increase in the weight loss during the storage time, which is mainly attributed to the senescence of the product and the loss of moisture content of the mushroom over time. In nano and nano + MAP packages, the percentage of weight loss in samples coated with chitosan was lower than that in uncoated samples in all days of the study, but its effect was not significant at a significance level of 1% or 5% (Table 2). Maghsudlo et al. (2012) similarly reported the effect of chitosan on changes in pistachio. The lower level of weight loss in coated samples can be attributed to the creation of a protective layer by chitosan coating on the product that acts as a barrier and prevents severe changes during the storage time.

**Table 2:** Analysis of variance of the effect of storage time, chitosan coating, and type of packaging on the physical properties of button mushroom

Source changes	*df*	Weight loss	*L* ^*^	Δ*E*	BI	pH	TSS
Storage time	5	8.12**	281.94**	88.53**	470.01**	0.78**	0.82**
Coating	1	0.22^ns^	32,932**	214.47**	30,285**	0.78**	0.34^ns^
Packaging	2	22.10**	268.80**	45.46**	6.89^ns^	2.35**	121.71**
Storage time × coating	5	0.08^ns^	73.80**	19.66**	129.68**	0.09**	2.39**
Storage time × packaging	10	2.46**	4.63^ns^	3.04^ns^	24.03^ns^	0.26**	2.04**
Coating × packaging	2	0.31^ns^	114.38**	4.48^ns^	375.90**	0.02**	0.42*
Storage time × coating × packaging	10	0.06^ns^	3.43^ns^	1.31^ns^	28.70^ns^	0.07**	0.74**
Error	72	0.33	11.35	3.40	19.74	0.00	0.14

Abbreviations: BI, Browning Index; *L* ^*^, Lightness index; ns, No significant difference; TSS, Total Soluble Solid; Δ*E*, Color change.

** A significant difference in the level of 1%.

* A significant difference in the level of 5%.

### 3.5 Color changes during the storage time

**Figure 4:**
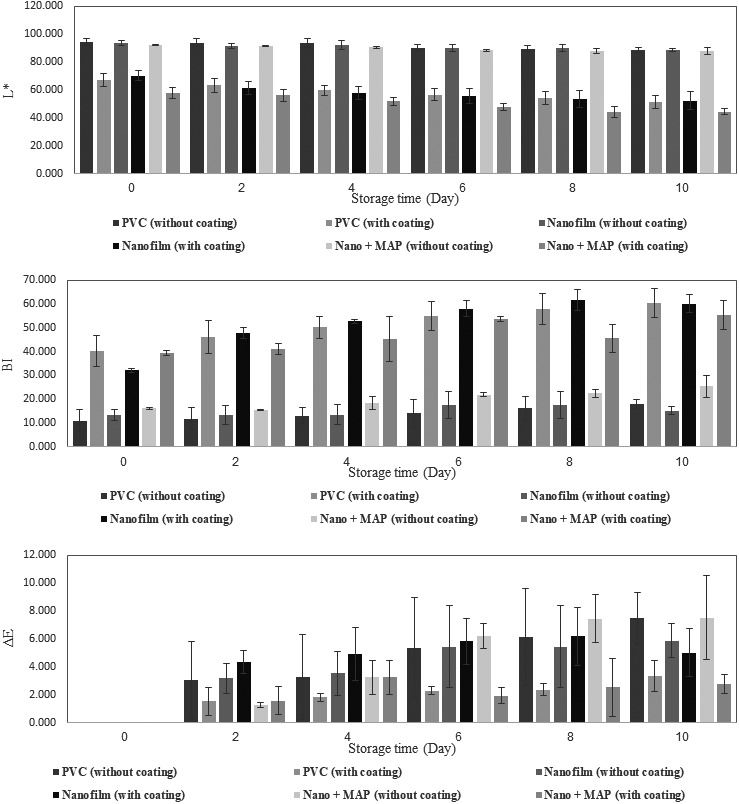
Changes in the color indices during the storage time

Color is one of the most important parameters determining the quality of white button mushroom. Therefore, the lightness index (*L* ^*^) as the most important color parameter in the mushroom, total color variation (Δ*E*) and browning index (BI) were assessed and measured during the storage time. The results showed a decrease in *L* ^*^ and an increase in Δ*E* and BI for all the samples during the storage time (Figure 4). Similar results on changes in color indices of some products are also reported by a number of researchers (El Enshasy, Elsayed, Aziz, & Wadaan, 2013; Rubilar et al., 2013). The results of analysis of variance of the samples showed that storage time, chitosan coating, and the interaction effect time × coating had a significant effect on all the three parameters at a significance level of 1%, while the triple interaction effect of time × coating × packaging did not have a significant effect on color indices at significance level of 1% or 5% (Table 2). The results showed that chitosan coating resulted in a severe reduction in *L* ^*^ and an increase in BI, as at the end of the storage time, the lowest level of *L* ^*^ and the highest level of BI were observed in the coated samples. In all the packages, the lowest level of browning was observed in uncoated samples which the lowest one was observed in samples placed in nanopackages (15.25) and the highest level of browning was observed in coated samples placed in conventional packages (60.46). There are two reactions that cause browning in mushrooms the first is called enzymatic browning and is caused by the release of the enzyme poly phenol oxidase (PPO) which cause brown and dark spots in the product (Asghari, Vaezi, & Farrokhzad, 2015). It reduces the quality of the mushroom and marketability of the product, and the second browning step called the Maillard reaction. Due to the presence of a spongy texture in button mushroom, the use of coatings solutions such as chitosan solution accelerates enzymatic activity and increases the browning index and decreases the lightness index during the storage time.

The results showed that type of packaging had no significant effect on changes in browning index at a significance level of 1%, while it has significant effects on changes in *L* ^*^ and Δ*E* at a significance level of 1% (Table 2). The results related to the assessment of the comparison of the mean values showed that among the three types of packaging, the nano + MAP packaging had a significant positive effect on *L* ^*^, and the lowest change (reduction) in *L* ^*^ until the end of the storage time (4.69%) was observed in uncoated samples in nano + MAP packaging. However, there was no significant difference between normal packaging and nanopackaging during the storage time. The results of the assessment of the mean interaction effects of time × coating × packaging showed that changes in time and type of packaging had no significant effect on L* in uncoated samples.

### 3.6 Percent open cap during the storage time

Cap opening increased in all treatments during the storage time, and the highest level of cap opening was observed in coated and uncoated samples packed with conventional film. After 10 days, the level of cap opening was 70% in samples in PVC and between 55% and 60% in samples in nano and nano + MAP packaging. The percent cap opening during the storage time is considered as the maturity index and indicates a decrease in moisture content in the product. The results showed that, similar to the weight loss (moisture reduction), cap opening was more significant in conventional packages than in other packages. It might be attributed to the relative insulation of nanofilm, which prevents the exit of water vapor from the package. The lowest amount of cap openings was observed in nano + MAP packages, which is also reported by some other researchers as well (Lopez Briones et al., 1992). High carbon dioxide and low oxygen levels have a positive effect on reducing cap opening and preventing the senescence of the product. The results of this study about cap opening were similar to results that reported by Oz et al. (2015).

### 3.7 Changes in chemical properties during the storage time

**Figure 5:**
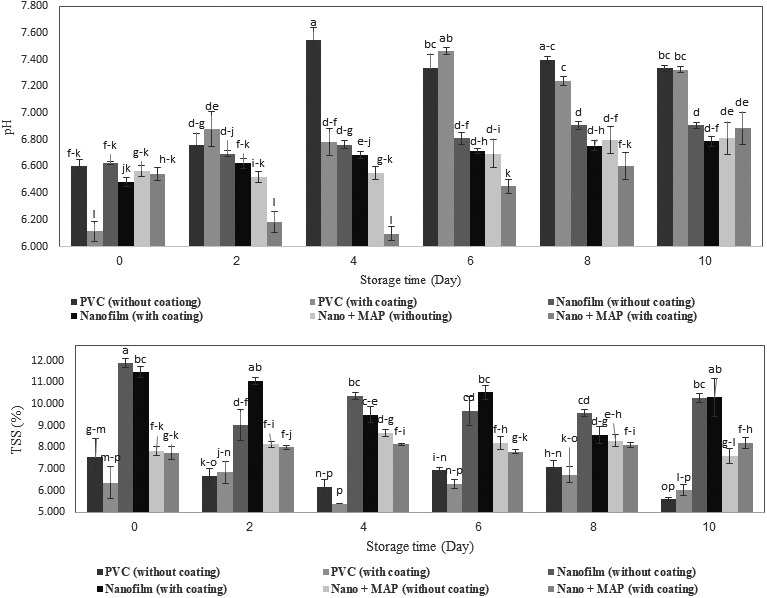
Changes in pH and TSS during the storage time

The results of analysis of variance showed that storage time, chitosan coating, type of packaging, and double and triple interaction effects had a significant effect on pH changes, at a significance level of 1% (Table 2). The changes in pH and the effect of the triple interaction effects of time × coating × packaging on mean pH and TSS values for all the samples are shown in Figure 5. The results of the assessment of the mean values of packaging and storage time showed that nano + MAP packaging had a significant effect on the control of pH at a significance level of 1%. In addition, during the storage time, the pH values were significantly different in all the days, except for the 6th and 8th day. The results showed that, at the end of the storage time, the changes in pH in uncoated samples were lower than that in coated samples in all the three types of packaging. On the other hand, the use of nano + MAP packaging prevented pH from being increased until the end of the storage time, as the lowest level of change was observed in uncoated samples in nano + MAP packages (3.57%); in fact, the use of this type of packaging helped to maintain the product properties until the end of the storage time. The highest level of change (increase) was observed in coated samples in PVC (16.52%). The barrier condition of nano and nano + MAP packaging and producing of CO_2_ causes to reduction the respiration rate of agricultural products, reduction of respiration rate postpones the aging; therefore, pH have controlled during storage (Mohammadpuor & Tajoddin, 2017). Controlling pH helps to preserve the initial conditions and keeps the product fresh after harvesting. In line with the results of our study, Tabatabaei Kloor et al. (2016) reported the effect of the type of packaging and control of respiration rate on the pH in tomato.

The changes in TSS did not have a regular trend during the storage time (Figure 5), but at the end of the storage time the amount of total soluble solids in all the treatments was lower than that in the first day. The lowest level of changes was observed in samples stored in nano + MAP packages (3.07%). The highest level of changes (34.52%) was observed in conventional packages, which led to destruction of cell walls and progressed senescence in the product. Asghari et al. (2015) reported a similar finding on the positive effect of nanofilm on the control and maintenance of total soluble solids during the storage time in sliced nectarine.

The results showed that in all conditions, the samples with chitosan coating had fewer changes in TSS, which might be due to the fact that chitosan creates a cover on the product and reduces the losses and stabilizes the soluble solids (Ghasemi Tvallaei et al., 2015); it also occurred for button mushroom, and TTS was controlled and maintained; however, it was not significant at a significance level of 1%. The dual and triple interaction effects in all the treatments, except for chitosan coating, had a significant effect on changes in TSS (Table 2). The results of the assessment of the compare mean and triple interaction effects of time × coating × packaging are shown in Figure 5. The assessment of the mean values of the main effects of the packaging showed that the changes in TSS were significant in all the three types of packaging at a significance level of 1%.

## 4 CONCLUSION

In this study, the effect of chitosan coating, type of packaging, and storage time on physical, chemical, and mechanical properties of button mushroom was investigated. The chitosan coating, type of packaging, storage period, and their double and triple interactions had a significant effect (at 1% and 5%) on most of the engineering parameters. The use of an oxygen barrier material with good permeability properties against carbon dioxide and low permeability to water vapor showed much better performance over the conventional PVC film; the use of MAP (10% O_2_ and 10% CO_2_) provided an extra benefit especially in terms of quality decay (e.g., in terms of overall appearance and weight loss), while chitosan coating had negative effects on some properties. As overall conclusion, experiments showed that using of nanofilm and nano + MAP condition had positive effects on preserving physical, chemical, and mechanical properties of white mushroom during storage and also can extend the shelf life of mushroom till 15th day. Therefore, use of nanofilm along with MAP (nano + MAP) is recommended for using in agricultural products packaging and food packaging industry especially for sensitive products like mushrooms. For other research, using of MAP is suggested, too, but should note that the gas composition must be selected based on the behavior of the samples. The approach presented in this study represents a promising alternative to conventional storage of white mushrooms. However, to confirm the importance of these results, additional tests will follow this first set of experiments. In particular, quantification of the respiration rate, enzyme assay (for the analysis of enzyme activity), malondialdehyde (MDA) content analysis (MDA is the main product of membrane lipid peroxidation), polyphenoloxidase (PPO) and peroxidase (POD) activity, antioxidant potential, and total phenolic content (all of them influencing the rate of enzymatic browning in the mushrooms) would be of help to unravel the basic mechanisms underlying the combined effect of temperature/packaging/MAP. Microbiological and sensory tests will instead provide the necessary information on safety and consumers' perception.

## CONFLICT OF INTEREST

All authors declare that there is no conflict of interest.

## ETHICAL STATEMENT

There was no human or animal testing in this study.
